# Predictive values of two frailty screening tools in older patients with solid cancer: a comparison of SAOP2 and G8

**DOI:** 10.18632/oncotarget.26147

**Published:** 2018-10-12

**Authors:** Chiara Russo, Chiara Giannotti, Alessio Signori, Michele Cea, Roberto Murialdo, Alberto Ballestrero, Stefano Scabini, Emanuele Romairone, Patrizio Odetti, Alessio Nencioni, Fiammetta Monacelli

**Affiliations:** ^1^ Department of Internal Medicine and Medical Specialties (DIMI), Section of Geriatrics, Genoa, Italy; ^2^ DISSAL, Section of Biostatistics, Department of Health Sciences, University of Genova, Genoa, Italy; ^3^ Hospital Policlinic San Martino, Oncological Surgery and Implantable Systems, Genoa, Italy

**Keywords:** aging, cancer, screening tool, geriatric assessment, frailty

## Abstract

**Objectives:**

Comprehensive Geriatric Assessment (CGA), the gold standard for detecting frailty in elderly cancer patients, is time-consuming and hard to apply in routine clinical practice. Here we compared the performance of two screening tools for frailty, G8 and SAOP2 for their accuracy in identifying vulnerable patients.

**Material and Methods:**

We tested G8 and SAOP2 in 282 patients aged 65 or older with a diagnosis of solid cancer and candidate to undergo surgical, medical and/or radiotherapy treatment. CGA, including functional and cognitive status, depression, nutrition, comorbidity, social status and quality of life was used as reference. ROC curves were used to compare two screening tools.

**Results:**

Mean patient age was 79 years and 54% were female. Colorectal and breast cancer were the most common types cancer (49% and 24%). Impaired CGA, G8, and SAOP2 were found in 62%, 89%, and 94% of the patients, respectively. SAOP2 had a better sensitivity (AUC 0.85, p<0.032) than G8 (AUC 0.79), with higher performance in breast cancer patients (AUC 0.93) and in patients aged 70-80 years (AUC 0.87).

**Conclusions:**

G8 and SAOP2 both showed good screening capacity for frailty in the cancer patient population we examined with SAOP2 showing a slightly better performance than G8.

## INTRODUCTION

The risk of cancer increases with age [1]. Already as of today, more than half of the patients who are newly diagnosed with cancer are older than 65 and this percentage is projected to increase to 70% by 2030 [2, 3]. This “oncogeriatric” population will benefit from the development of innovative therapies or surgical procedures, which will, however, require specific clinical management [4].

Despite the increasing rate of elderly cancer patients, there is a major gap of knowledge on how to properly stratify older patients according to their biological status, to be able to recommend the most appropriate type of treatment in a personalized fashion.

The Comprehensive Geriatric Assessment (CGA) is a multidimensional, interdisciplinary evaluation that leads to the identification and stratification of patients’ clinical vulnerability.

According to the International Society of Geriatric Oncology (SIOG), the CGA remains the gold standard for defining the presence and or the degree of frailty in elderly cancer patients; its widespread use is recommended, particularly since the CGA was shown to improve patient overall survival, functional status, and quality of life [5, 6]. In geriatric oncology frailty's evaluation is mainly focused on person's ability to tolerate cancer treatment; frailty is also associated with a worse quality of life.

However, the CGA is time and resource-consuming and requires the expertise of geriatricians who are not always available in standard cancer clinics. It remains poorly incorporated in routine clinical practice. Thus, consensus guidelines from the National Comprehensive Cancer Network (NCCN) [7], the European Organization for Research and Treatment of Cancer (EORTC) [8], and the SIOG [9], consider a “two-step approach” as a reasonable strategy, where the first step involves a geriatric screening test to identify patients who are at high risk of being frail and the second step foresees a complete CGA to be performed by geriatricians [1, 10–13].

Since the 2005 SIOG guidelines, a total of 17 different tools have been studied in 44 different trials to evaluate the best screening test in oncogeriatrics [9]. These include G8, Oncogeriatric screen (OGS), Abbreviated Comprehensive Geriatric Assessment (aCGA), Senior Adult Oncology Program (SAOP) 2, Gerhematolim, the Vulnerable Elders Survey-13 (VES-13) and Flemish version of the Triage Risk Screening Tool (fTRST) [9].

So far, although the G8, the VES-13 and fTRST tools have shown the best clinometric properties in elderly patients [9], there remains a substantial uncertainty as to which test most adequately identifies frailty in at risk older cancer populations [14].

The purpose of this study was to define the accuracy with which two geriatric screening tools, G8 and SAOP2 identify frail oncogeriatric patients, who are ultimately candidates to receive a CGA.

## RESULTS

### Patients’ clinical characteristics

**Figure 1:**
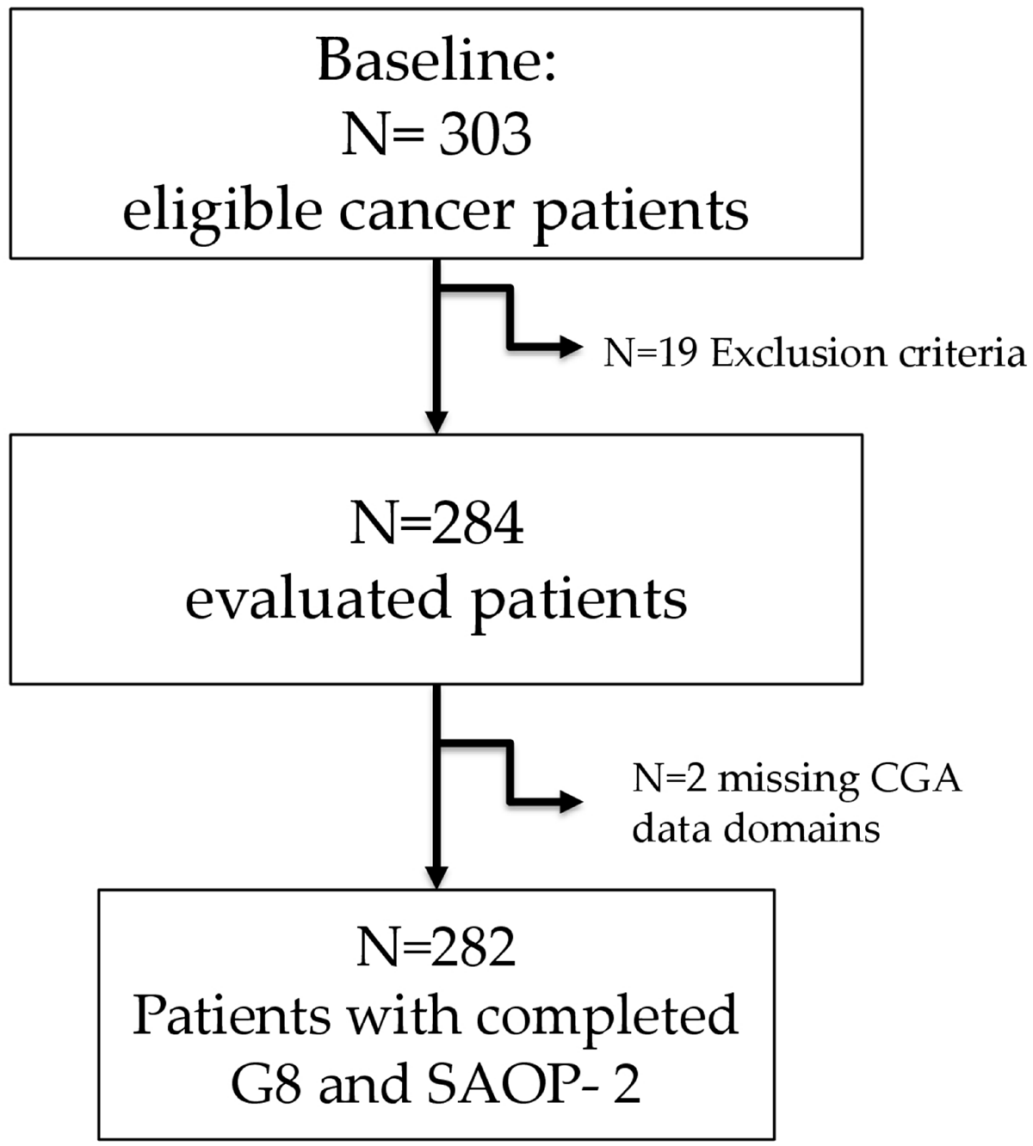
Study sample design

Three hundred three eligible cancer patients were evaluated at the Ospedale Policlinico San Martino in Genoa, Italy, between January 2015 and May 2017. For two patients, clinical data collection was not completed and therefore they were not included in the study. Nineteen patients did not meet the inclusion criteria. Thus, 282 eligible patients were evaluated in the study (Figure 1). Mean patient's age was 79.02 years ± 5.87 (range, 65-93 years) and about 40% of the patients were >80 years old. 54% of the patients were female and 46% were male. Colorectal cancer and breast cancer were the most common types of neoplasms for which patients were being treated, accounting for 50% and 24% of the patients, respectively.

Patients’ clinical characteristics, screening tools and CGA assessment are illustrated in Table 1 and 2.

**Table 1:** Patients’ demographic and clinical characteristics

VARIABLE	N
***DEMOGRAPHICS***	
*GENDER:*	
*FEMALE*	*152*
*MALE*	*130*
*MEAN AGE*	*79.02 ± 5.87 (range 65–93 YEARS)*
*AGE*	
*< 69*	*14*
*70 - 80*	*156*
*81 - 89*	*101*
*> 90*	*11*
***CANCER TYPE***	
*COLORECTAL*	*138*
*GASTRIC AND OESOPHAGEAL*	*15*
*PANCREAS AND BILIAR TRACT*	*4*
*HEAD AND NECK*	*12*
*BREAST*	*68*
*PROSTATE*	*11*
*GYNAECOLOGICAL*	*11*
*RENAL AND BLADDER*	*11*
*LUNG*	*3*
*OTHERS*	*9*
***DISEASE STAGE***	
*NON-METASTATIC DISEASE*	*186*
*METASTATIC DISEASE*	*14*
*UNCLASSIFIED*	*82*

**Table 2:** G8, SAOP2 screening tools, CGA assessment with frequency of elders who were categorized as impaired in each domains of CGA

*SCREENING TOOLS AND* *CGA ASSESSMENT*	CLINICAL DOMAIN	CUT-OFF ^*^	IMPAIRED %	MEAN SCORE ± SD
*G8*		≤14	74	*12.23 ± 2.75*
*SAOP2*		>2	78	*2.73 ± 1.32*
*MMSE*	COGNITIVE STATUS	<24	20	*26.65 ± 3.88*
*CDT (SCHULMAN)*	COGNITIVE STATUS	≥ 3	48	*2.54 ± 1.40*
*MNA*	NUTRITIONAL STATUS	< 23	44	*22.62 ± 3.91*
*IADL*	FUNCTIONAL STATUS	≤ 7	39	*6.83 ± 2.02*
*BARTHEL INDEX*	FUNCTIONAL STATUS	< 50	3,5	*95.98 ± 11.64*
*CIRS* *COMORBIDITY* *SEVERITY*	COMORBIDITY	>3	63	*4.23 ± 1.83* *1.96 ± 0.38*
*N° OF DRUGS*	-	≥ 3	57	*4.48 ± 2.75*
*GDS*	PSYCHOLOGICAL STATUS	≥ 5	30	*3.92 ± 3.34*
*TINETTI SCALE*	POSTURAL STABILITY	≤ 18	16,5	*24.02 ± 5.74*
*MORSE SCALE*	RISK OF FALL	≥ 25	29	*23.44 ± 19.94*
*GIJON SCALE*	SOCIAL STATUS	≥ 10	35	*8.77 ± 2.29*
*SF-36*	*QoL*			*0.68 ± 0.21*
CUT OFF CGA^**^		≥ 3	62	*3.77 ± 2.56*

Abbreviations: SAOP2: Senior Adult Oncology Program (SAOP) 2; MMSE: Mini Mental State Examination; CDT: Clock Drawing Test; MNA: Mini Nutritional Assessment ; I-ADL: Instrumental Activities of Daily Living; CIRS: Cumulative Illness Rating Scale; GDS: Geriatric Depression Scale; SF-36: Short Form 36; QoL: Quality of life; CGA: Comprehensive Geriatric Assessment.

^*^ Cut-off score.

^**^ cumulative number of impaired CGA domains.

Overall, 175 out of 282 patients (62%) showed problems in at least 3 CGA clinical domains, thus resulting as frail. This clinical vulnerability was mainly characterized by multimorbidity, initial functional decline and malnutrition risk (Table 2). In addition, patients reported a poor perception of the quality of life according to Short Form 36 (SF-36).

Notably, based on the CGA assessment, a G8 impairment mostly reflected an increased malnutrition risk (Mini Nutritional Assessment-MNA) (U 642, p<0.05) (Table 3).

**Table 3:** Comparison of CGA domains in patients aged between 70 and 80 years with impaired G8 and SAOP2, respectively

CLINICAL DOMAIN AND TOOL MEAN SCORE (±SD)	N 156	G8 ≤ 14 N 114	SAOP ≥ 2 N 131	P value ^b^
**COGNITIVE STATUS** **MMSE**	27.29±2.95	26.93±3.25	26.85±3.16	ns
**FUNCTIONAL STATUS** **BARTHEL**	97.01±9.48	95.75±11.47	96.03±10.75	ns
**FUNCTIONAL STATUS** **IADL**	7.16±1.67	6.83±1.95	6.91±1.86	ns
**PHYSICAL PERFORMANCE** **TUG**	10.15±4.27	10.80±4.87	10.77±4.60	ns
**NUTRITIONAL STATUS** **MNA**	22.53±4.91	20.85±4.76	21.76±4.88	U 642, p<0.05
**PSYCHOLOGICAL STATUS** **GDS**	3.89±3.44	4.45±3.70	4.54±3.62	ns
**SOCIAL STATUS** **GIJON SCALE**	8.53±2.36	8.99±2.44	9.02±2.39	ns
**COMORBIDITY** **CIRS CI**	4.05±1.83	4.43±1.91	4.40±1.85	ns
**QOL** **SF-36**	0.69±0.21	0.65±0.22	0.65±0.22	ns

^b^ non-parametric Mann–Whitney U test.

Abbreviations: CGA: Comprehensive Geriatric Assessment; SAOP2: Senior Adult Oncology Program (SAOP) 2; MMSE: Mini Mental State Examination; I-ADL: Instrumental Activities of Daily Living; TUG: Timed “Up & Go” test; MNA: Mini Nutritional Assessment; GDS: Geriatric Depression Scale; CIRS: Cumulative Illness Rating Scale; CI: Co-morbidity Index; QoL: Quality of life; SF-36: Short Form 36.

Frail patients (based on CGA assessment) scored positive according to G8 in 89% and to SAOP2 in 94% of the cases, respectively (Table 4).

**Table 4:** Comparison between G8 and SAOP2 diagnostic accuracy with reference to the gold standard CGA

	Sensitivity	Specificity	PPV	NPV
G8	89%	49.5%	73.9%	73.6%
SAOP2	94%	46.9%	74%	81%

Abbreviations: CGA: Comprehensive Geriatric Assessment; SAOP2: Senior Adult Oncology Program (SAOP) 2; PPV: positive predictive value; NPV: negative predictive value.

The comparison between G8 and SAOP2 diagnostic accuracy showed that SAOP2 had fair specificity, lower than G8.

**Figure 2:**
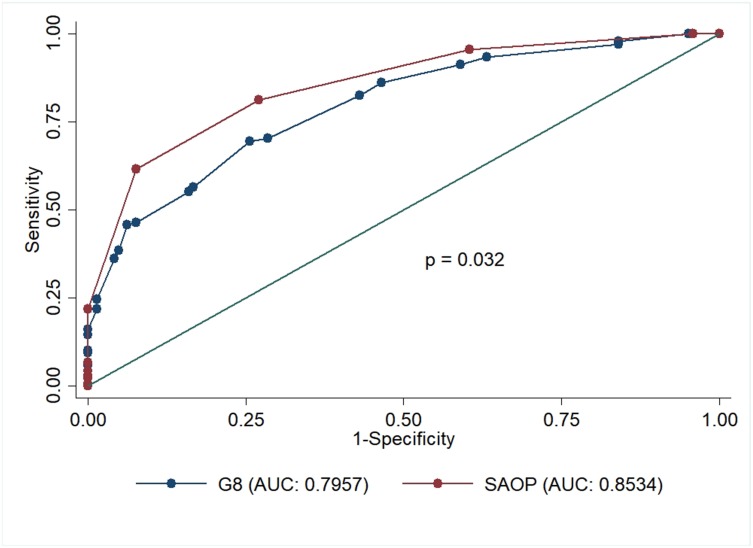
ROC curve diagnostic accuracy comparison between G8 and SAOP2 screening tools with reference to CGA Abbreviations: ROC: Receiver operating characteristic; SAOP2: Senior Adult Oncology Program (SAOP) 2; CGA: Comprehensive Geriatric Assessment.

A pairwise comparison between SAOP2 (n=282; AUC 0.85; 95% CI: 0.0215-0.81130) and G8 (n=282; AUC 0.79; 95% CI: 0.0260-0.74478) with reference to CGA using ROC curves showed a higher accuracy in differentiating patients with abnormal CGA for the SAOP2 screening tool (p<0.032) (Figure 2).

The diagnostic accuracy of both screening tools (with reference to CGA) was further assessed, separately, in patients with a diagnosis of breast cancer (n=68) versus patients with a diagnosis of colorectal cancer (n=138).

**Figure 3:**
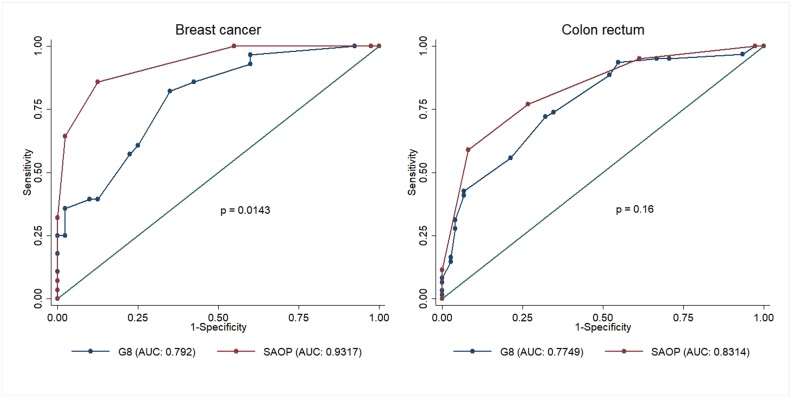
ROC curve comparison of G8 and SAOP2 screening tools in patients with breast cancer and colon rectal cancer respectively Abbreviations: ROC: Receiver operating characteristic; SAOP2: Senior Adult Oncology Program (SAOP) 2.

The SAOP2 showed higher accuracy in predicting patients’ clinical vulnerability in breast cancer patients (n= 68; AUC 0.93; 95% CI: 0.87822-0.98518) as compared to the G8 (n=68; AUC 0.79; CI: 0.68674-0.89719) (p<0.014). Conversely, a comparison between SAOP2 and G8 in colorectal cancer patients did not show any difference in the ability to detect frail patients (p<0.160) (Figure 3).

In addition, in patients aged between 70 and 80 years, SAOP2's diagnostic accuracy (n=156; AUC 0.87; 95% CI 0.82061-0.92710) was higher as compared to G8's accuracy (n=156; AUC 0.77; 95%CI 0.70043-0.84865) (p<0.015).

**Figure 4:**
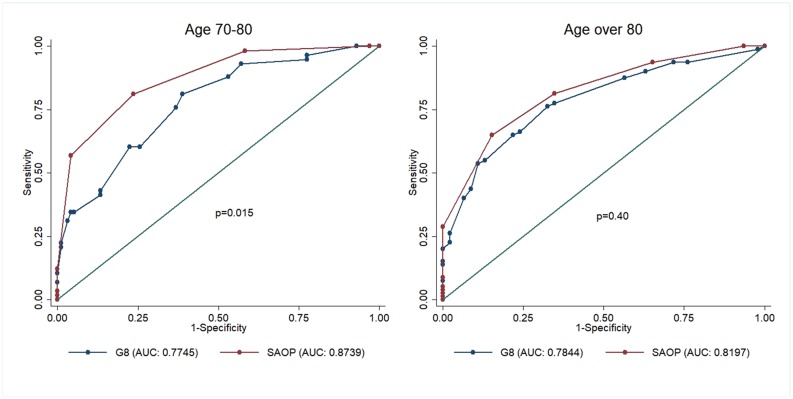
ROC curve comparison of G8 and SAOP2 screening tools in patients aged 70- 80 years and in patients > 80 years Abbreviations: ROC: Receiver operating characteristic; SAOP2: Senior Adult Oncology Program (SAOP) 2.

Notably, both screening tools failed to accurately detect frailty in patients aged >80, with a high rate of false positive results (p<0.40) (Figure 4).

## DISCUSSION

The integration of CGA in clinical practice could help oncologists tailor clinical decisions based on the elderly patient's actual fitness. Currently, a two-step approach is recommended. Nevertheless, the best screening tool to be applied in the clinic remains to be defined [14].

To the best of our knowledge, this is the first study to directly compare the performance of two commonly applied frailty screening tools, G8 and SAOP2, in an older cancer population.

Clearly, a high sensitivity and specificity are both desired properties of an oncogeriatric-screening tool, to limit the number of fit patients who unnecessarily undergo CGA assessment. These clinometric properties also ensure that frailty is properly recognized, thus avoiding that vulnerable subjects are over treated and exposed to the risk of treatment toxicity [9].

Our data show that the SAOP2 has higher diagnostic accuracy as compared to the G8, especially in oncogeriatric patients who are <80 years. Conversely, both SAOP2 and G8 showed adequate sensitivity at the expense of specificity in patients older than 80 years. Furthermore, our results indicate that the SAOP2 screening tool has better screening performance than the G8 in breast cancer patients, but not in patients diagnosed with colorectal cancer.

Evidence is accumulating on the role of G8 screening tool in detecting vulnerable patients, even if with heterogeneous results [9, 15]. The ONCODAGE multicentre study [16] has validated the G8 for the identification of older cancer patients eligible for CGA assessment: sensitivity varied according to tumour site and stage (head and neck cancer 94%; colon cancer 88%; metastatic stages 87%). Further, Kenis et al [13] has shown high sensitivity and moderate negative predictive value of the G8 tool in elders with metastatic breast and colorectal cancer.

Conversely, the comparison among fTRST, G8, and Groningen Frailty Index in elderly cancer patients [17] has resulted in the higher diagnostic accuracy of fTRST (sensitivity 92%) compared to G8 (sensitivity 80%). Baitar et al [18] has confirmed the higher accuracy of G8 tool in identifying vulnerable cancer patients with prevalent malnutrition.

Furthermore, in neck and head cancer patients, the G8 tool has shown better sensitivity, compared to VES-13 [19], and, similarly, Liu et al [20] has reported G8 higher sensitivity in patients with local colorectal cancer, upper digestive, hepatic tumour and in the metastatic group. In keeping with that, Pottel et al [21] has underpinned more impaired G8 scores in patients with advanced cancer, compared to early stage cancer patients.

Thus, it is plausible that subjects with more advanced cancer, and particularly with gastrointestinal and head/neck tumour are frequently comorbid for malnutrition [22], due to the intense inflammatory response associated with anorexia and cachexia [23], which can lead to progressive loss of skeletal muscle mass and worsen impairment of function [24]. Malnutrition has been associated with reduced ability to tolerate anti-cancer therapy, increased severe dose-limiting toxicities, lesser response rates, worse quality of life, decline in performance status, and shorter survival outcomes [25]. Thus, malnutrition turns to play a key relevant weight in informing G8 impairment.

Indeed, G8 screening tool incorporates most of the MNA items and the fact that MNA was not designed to specifically detect an abnormal CGA may probably explain the lack of specificity of the G8 as a screening instrument [15].

Conversely, in haematological malignancies, the G8 tool didn't adequately discriminate unfit subjects eligible for CGA [26], showing moderate diagnostic accuracy. Hamaker et al [27] has indicated lower sensitivity but better specificity (respectively 69% and 79%) of G8 screening performance due to potential higher prevalence of underdiagnosed geriatric syndromes.

This scientific background may count for the higher accuracy of SAOP2 screening in intercepting vulnerability in breast cancer patients compared to colorectal patients, as observed in the present study.

Thus, different cancer types and stages may have a different weighed impact on screening performance and overall diagnostic accuracy and the use of several validated CGA instruments and cut-off values may also add methodological biases, affecting results reliability.

Fewer evidence has shown the diagnostic accuracy of SAOP2 screening tool in different cancer population and clinical settings. However, SAOP2 tool [28] has shown adequate clinometric properties with reference to the standard geriatric assessment (sensitivity of 100% and a specificity of 40%) [29], including the assessment of key relevant issues for cancer related outcomes, such as social vulnerability, depression, quality of life and perceived health status.

Lower perceived social support is generally associated with higher depressive symptoms and lower quality of life [30, 31], especially in cancer patients compared to the general population [30, 32]. Thus, social vulnerability represents a key factor for patient's compliance and the effectiveness of chemotherapy regimens [33, 34]. It has also been shown that social vulnerability and frailty are related but distinct clinical constructs [35] and that the former was a significant predictor of mortality and disability, regardless of patients’ frailty [36, 37].

Originally, the present findings indicate SAOP2 better performance in patients aged 70-80 years and support the diagnostic inaccuracy of oncogeriatric screenings (higher false positive screening results) in over octogenarian patients. The biological aging involves a loss of homeostasis with enhanced vulnerability to environmental stressors (surgical interventions or chemotherapy) [33, 38] that may exceed patients’ threshold homeostenosis, precipitating a frailty trajectory [39]. Moreover, it is likely that the highly individualized trajectory of frailty could affect the discriminative power of these screening tools, especially in the oldest old (>85 years) populations [40, 41]. In turn, the time-saving potential of screening may outweigh the risk of incorrectly identifying patients, delivering inappropriate care [41].

On the basis of our results, G8 performance seems to be outweighed by malnutrition risk in cancer patients between 70 and 80 years. Conversely, SAOP2 tool did not show any correlation with GCA domains.

Few studies have addressed the association between single geriatric domains, with reference to CGA assessment, and oncogeriatric screening performance [41]. Namely, Hamaker et al. has shown that G8 had strong predictive ability for malnutrition, but lower predictive value for geriatric conditions. In addition, VES-13 had a fair predictive value for cognitive disorders, impaired mobility, and malnutrition [41]. The association of screening tools with social support, showed a very low diagnostic accuracy (VES-13 sensitivity 33%; specificity 46%) [41–44].

In our population, SAOP2 was not statistically associated with social domains even an association trend with poorer perceived health status and lack of support was observed. This may be due to the partial diagnostic accuracy of the used Gijon Scale; in turn, the social vulnerability index (SVI) [36], may be the most appropriate tool in elderly cancer patients. However, the lack of any Italian validation hampers the feasibility of such a tool in intercepting patients’ social vulnerability.

Even preliminary in nature, SAOP2 tool seems to better predict clinical vulnerability, especially at the earlier stages of cancer. This is particularly true in ‘’younger’’ patients with two wide prevalent cancer types.

The present study has some limitations. First, non-metastatic colorectal and breast cancer were strongly represented while patients with progression/relapse of the disease were systematically excluded. Thus, stratification of patients with different cancer types and stages is still needed. Moreover, the single centre population may represent a potential bias selection.

Notwithstanding that, the strength of the study lies on the real-world assessment of an oncogeriatric population, with the direct comparison of G8 and SAOP2 screening accuracy. Assessing this aspect would warrant more sophisticated study designs, including the feedback of a GA team, the appropriateness of the referral and the time frame of clinical interventions. In line with that, the prospective nature of the present study will help the understating of outcome measures in terms of service utilization, geriatric referrals, complications, functional independence and survival variables.

Comparing sensitivity and specificity to CGA has the advantage of feasibility in a study. However, it only indirectly addresses the question of how useful the tool is for selecting patients. Therefore, which screening tool could best suit the older cancer populations is a matter of debate. The inclusion of frailty indicators and biological markers may add knowledge to this intriguing field and is part of the observational study.

Eventually, further research is needed to optimize the use of SAOP2 screening tool. On the basis of its multidisciplinary nature and the inclusion of key relevant issues like social vulnerability, health perceived status and quality of life, it has great potential of defining clinical pathways, targeting the quality of life and the quality of care in this outgrowing number of cancer patients [15].

## MATERIALS AND METHODS

### Study design and population

This prospective study was performed at the Ospedale Policlinico San Martino, Genoa, Italy, from January 2015 to May 2017.

Inclusion criteria were all patients over 65 years with a first diagnosis of solid tumour, who qualified for surgery and/or chemotherapy, adequate understanding of the Italian language and ability to sign an informed consent.

Exclusion criteria were: palliative care patients; severe dementia or pre-existing major neurological and/or psychiatric disorders.

The study was approved by the ethical committee of the participating hospital, and written informed consent was obtained by all subjects or their next to kin.

Patients were simultaneously tested for G8 and SAOP2 questionnaire and comprehensive geriatric assessment, before oncological treatment (surgery, neoadjuvant or adjuvant chemotherapy), by an expert geriatrician.

First visit also included the Short Form 36 [45, 46] to assess quality of life.

Demographic data (age, gender), tumour characteristics (site, local or metastatic), proposed chemotherapy and/or surgery, geriatric recommended clinical interventions were also collected.

### Test methods

#### G8 screening tool

The G8 screening tool was developed to identify elderly unfit cancer patients, eligible for geriatric assessment.

The G8 test consists of the following eight items: chronological age (<80, 80–85, >85 years) and seven clinical items including the Mini Nutritional Assessment, a questionnaire dealing with food intake, weight loss, mobility, neuropsychological comorbidity, body mass index, prescription drug, and self-perception of health status [1, 22, 40].

The total score can range from 0 to 17. A score of ≤14 is considered abnormal, indicating a clinical vulnerability profile. (Supplementary Appendix 1)

The G8 was compared in terms of clinometric properties with CGA in eight different studies, that cumulatively included 3816 patients [13, 16–20, 40, 47]. Sensitivity ranged from 65% to 92%, specificity ranged from 3% to 75% and negative predictive value (NPV) from 8% to 78% [9].

### Senior adult oncology program (SAOP) 2

The SAOP2 screening tool was developed by the multidisciplinary clinical team of the SAOP at Moffitt Cancer Centre [10]. In addition to functional status, depression, and cognitive screening, the tool includes the assessment of health-related quality of life, self-rated health, falls, nutrition, sleep, multiple medications, and social issues (drug payment and reimbursement and caregiver availability) (Supplementary File 2) [10].

If 1 item is impaired, the respective specialist is called in, with potential secondary referral to other team members. If several items are impaired, the multidisciplinary team is called along with the geriatric referral for CGA assessment. SAOP2 is a sensitive tool, with low internal specificity, addressing the importance of a multidisciplinary team approach [28, 29, 48].

### Comprehensive geriatric assessment (CGA)

An expert geriatrician administered the CGA assessment in an average time of 50 minutes. It evaluates the following tools to assess several clinical domains (Table 5): cognitive status (Mini Mental State Examination, MMSE [49] and Clock Drawing Test, CDT [50]), psychological status (Geriatric Depression scale, GDS 15 items [51]), functional status (Instrumental Activities of Daily Living, IADL, of Lawton [52] and Barthel Index [53]), postural stability (Tinetti Scale [54]), risk of falls (Morse Scale [55]), physical performance (Timed “Up & Go” test, TUG [56, 57]), nutritional status (Mini Nutritional Assessment [58]), social vulnerability (Gijon Scale [59]), physical burden of illness (Cumulative Illness Rating Scale, CIRS: Illness Severity Index-SI, and Co-morbidity Index-CI [60, 61]). Patients were categorized as impaired if the CGA ≥3 deficits [62].

**Table 5:** CGA assessment, clinical domain and cut-offs

Tool	CLINICAL DOMAIN	NUMBER OF ITEMS	RANGE	CUT-OFF ^*^
IADL	FUNCTIONAL STATUS	8	0-8	≤ 7
BARTHEL	FUNCTIONAL STATUS	10	0-100	< 50
MORSE SCALE	RISK OF FALL	6	0-125	≥ 25
TINETTI SCALE	POSTURAL STABILITY	16	0-28	≤ 18
CIRS SEVERITY COMORBIDITY	COMORBIDITY	19	0-37 0-5 0-13	>3
MMSE	COGNITIVE STATUS	7	0-30	<24
CDT	COGNITIVE STATUS	1	1 -6	≥ 3
GDS	PSYCHOLOGICAL STATUS	15	0-15	≥ 5
MNA	NUTRITIONAL STATUS	18	0-30	< 23
NRS	PAIN	1	0-10	≥ 3
GIJON SCALE	SOCIAL STATUS	5	5-25	≥ 10
CGA	-	-	-	≥ 3

Abbreviations: I-ADL: Instrumental Activities of Daily Living; CIRS: Cumulative Illness Rating Scale; SI: Illness Severity Index; CI: Co-morbidity Index; MMSE: Mini Mental State Examination; CDT: Clock Drawing Test Shulman; GDS: Geriatric Depression Scale; MNA: Mini Nutritional Assessment; NRS: Numeric Rating Scale; CGA: Comprehensive Geriatric Assessment.

^*^ Cut-off score.

Pain was assessed using the Numeric Rating Scale (NRS) [63, 64]. Polypharmacy was also collected.

### Outcome

The primary outcome was to assess the diagnostic accuracy of SAOP2 and G8 screening tool, with reference to CGA, in detecting patient's clinical vulnerability.

### Statistical analysis

The descriptive analysis for quantitative variables was expressed as mean and standard deviation (SD) or median and interquartile range (IQR).

Sensitivity and specificity of both screening tools were calculated using the pre-specified cut-offs from literature.

Further receiver operating characteristic (ROC) curves were used to compare G8 and SAOP2 screening tools.

If present, indeterminate results were considered as false-positive or false-negative and incorporated into the final analysis. For example, an indeterminate result in a patient found to be frailty according to CGA was considered to have had a negative test result.

Area under the curves (AUC) with 95% CI were reported. AUCs were compared using chi-square test.

A non-parametric Mann–Whitney test was used to compare two variable.

A p-value <0.05 was considered statistically significant.

Stata (v.14; function “roccomp”; StataCorp) was used for the computation.

Collection and assembly of data: CR, CG, SS, ER, RM, AB.

Data analysis and interpretation: CR, CG, FM.

Statistical analysis: CG, AS, FM.

Manuscript writing: CR, CG, FM, AN.

## SUPPLEMENTARY MATERIALS FIGURES AND TABLES







## Abbreviations

CGA: Comprehensive Geriatric Assessment
SIOG: International Society of Geriatric Oncology
NCCN: National Comprehensive Cancer Network
EORTC: European Organization for Research and Treatment of Cancer
SAOP2: Senior Adult Oncology Program (SAOP) 2
OGS: Oncogeriatric screen
aCGA: Abbreviated Comprehensive
VES-13: Vulnerable Elders Survey-13
fTRST: Flemish version of the Triage Risk Screening Tool
GFI: Groninger Frailty Index
IR: Rockwood Index
SF36: Short Form 36
MNA: Mini Nutritional Assessment
GA: geriatric assessment
NPV: negative predictive value
MMSE: Mini Mental State Examination
CDT: Clock Drawing Test Shulman
GDS: Geriatric Depression Scale
I-ADL: Instrumental Activities of Daily Living
TUG: Timed “Up & Go” test
CIRS: Cumulative Illness Rating Scale
SI: Illness Severity Index
CI: Co-morbidity Index
NRS: *Numeric Rating Scale*
SVI: social vulnerability index
CTCAE: National Cancer Institute Common Terminology Criteria for Adverse Events
SD: standard deviation
IQR: median and interquartile range
AUC: Area under the curves
ROC: Receiver operating characteristic.
