# Supplementary material for: Predictive values of two frailty screening tools in older patients with solid cancer: a comparison of SAOP2 and G8
Source: Oncotarget. 2018 Oct 12;9(80):35056–68. doi: 10.18632/oncotarget.26147 (PMC6205549; doi:10.18632/oncotarget.26147)
Supplement: Supplementary file 1 [file oncotarget-09-35056-s001.pdf]

Predictive values of two frailty screening tools in older patients with solid cancer: a comparison of SAOP2 and G8

SUPPLEMENTARY MATERIALS

Supplementary Appendix 1: The G-8 questionnaire

See Supplementary File 1

Supplementary Appendix 2: Senior Adult Supplement Screening Questionnaire SAOP2

See Supplementary File 2
