# Supplementary material for: Predictive values of two frailty screening tools in older patients with solid cancer: a comparison of SAOP2 and G8
Source: Oncotarget. 2018 Oct 12;9(80):35056–68. doi: 10.18632/oncotarget.26147 (PMC6205549; doi:10.18632/oncotarget.26147)
Supplement: Supplementary file 2 [file oncotarget-09-35056-s002.pdf]

# G8 questionnaire

| | Items | Possible answers (score) |
|---|---|---|
| **A** | Has food intake declined over the past 3 months due to loss of appetite, digestive problems, chewing or swallowing difficulties? | 0 : severe decrease in food intake |
| | | 1 : moderate decrease in food intake |
| | | 2 : no decrease in food intake |
| **B** | Weight loss during the last 3 months | 0 : weight loss > 3 kg |
| | | 1 : does not know |
| | | 2 : weight loss between 1 and 3 kgs |
| | | 3 : no weight loss |
| **C** | Mobility | 0 : bed or chair bound |
| | | 1 : able to get out of bed/chair but does not go out |
| | | 2 : goes out |
| **E** | Neuropsychological problems | 0 : severe dementia or depression |
| | | 1 : mild dementia or depression |
| | | 2 : no psychological problems |
| **F** | Body Mass Index (BMI (weight in kg) / (height in $m^2$) | 0 : BMI < 19 |
| | | 1 : BMI = 19 to BMI < 21 |
| | | 2 : BMI = 21 to BMI < 23 |
| | | 3 : BMI = 23 and > 23 |
| **H** | Takes more than 3 medications per day | 0 : yes |
| | | 1 : no |
| **P** | In comparison with other people of the same age, how does the patient consider his/her health status? | 0 : not as good |
| | | 0.5 : does not know |
| | | 1 : as good |
| | | 2 : better |
| | Age | 0 : >85 |
| | | 1 : 80-85 |
| | | 2 : <80 |
| | **TOTAL SCORE** | **0 – 17** |
