# Supplementary material for: Predictive values of two frailty screening tools in older patients with solid cancer: a comparison of SAOP2 and G8
Source: Oncotarget. 2018 Oct 12;9(80):35056–68. doi: 10.18632/oncotarget.26147 (PMC6205549; doi:10.18632/oncotarget.26147)
Supplement: Supplementary file 3 [file oncotarget-09-35056-s003.pdf]

Senior Adult Supplement Screening Questionnaire
SAOP2

1. If it was necessary, is there someone who could help take care of you? **[ ] Yes [ ] No**
2. Do you feel sad more days than Not? **[ ] Yes [ ] No**
3. Have you lost interest in things you used to enjoy (hobbies, food, sex, being with friends/family)? **[ ] Yes [ ] No**
4. On a scale of 1 to 10, rate your *present* quality of life (10 is the best life, 1 is the worst)

   1 2 3 4 5 6 7 8 9 10

   Worst ***Best***
5. On a scale of 1 to 10, rate your *present* overall health (10 is excellent, 1 is poor)

   1 2 3 4 5 6 7 8 9 10

   [Poor] [Fair] [Good] [Excellent]
6. Activities of Daily Living:

Can you dress yourself completely?	[ ] Yes	[ ] Yes but with help	[ ] No
Can you feed yourself?	[ ] Yes	[ ] Yes but with help	[ ] No
Do you use a cane, walker, or wheelchair?	[ ] Yes	[ ] Yes occasionally	[ ] No
Do you need help to get out of bed/chair?	[ ] Yes	[ ] Yes but with help	[ ] No
Are you incontinent of urine?	[ ] Yes	[ ] Occasionally	[ ] No
Do you need help taking a shower or a bath?	[ ] Yes	[ ] Occasionally	[ ] No
Have you tripped or fallen in the past year?	[ ] Yes		[ ] No
Are you able to drive?	[ ] Yes	[ ] Have Never Driven	[ ] No
Are you able to prepare your own meals?	[ ] Yes	[ ] Yes but with help	[ ] No
Are you able to go shopping?	[ ] Yes	[ ] Yes but with help	[ ] No
Can you take care of your finances?	[ ] Yes	[ ] Yes but with help	[ ] No
Can you use the telephone?	[ ] Yes	[ ] Yes but with help	[ ] No
Do you remember to take your medications?	[ ] Yes	[ ] Yes but with help	[ ] No

7. Have you lost 5 or more pounds in the past 6 months <u>without dieting</u>? [ ] Yes [ ] No
8. Has your appetite decreased in the last 3 months? [ ] Yes [ ] No
9. Has there been a change in the *types* of foods you are able to eat? [ ] Yes [ ] No
10. Are you always able to pay for your prescription medications? [ ] Yes [ ] No
11. Do you feel you are sleeping well? [ ] Yes [ ] No

______________________________ Signature of Patient or Legal Representative

______________________________ Date

______________________________ Signature of Person Completing Form (if not patient)

______________________________ Relationship to Patient

Please stop here. Thank you!

PATIENT NAME: ______________________________

DATE OF BIRTH: ______________________

MR#: ______________________________

MOFFITT CANCER CENTER®

16952-1-004 10/10

Senior Adult Supplement Screening Questionnaire
SAOP2

***I am going to name 3 objects (**pencil, truck, book**) and ask you to repeat them now and a few minutes from now to test your memory.

12. Spell the word "**clown**" backwards. n-w-o-l-c — 5 points_____

13. What is today's date and day?
Month._______ Date_______Yr.______, Day__________ — 4 points_____

14. Can you repeat the 3 objects I mentioned earlier? [ ] 1 [ ] 2 [ ]3 — 3 points_____

Total _____

15. How many medications/herbals/vitamins are you taking? ____________ [ ] None

**Additional Information**			
Eastern Cooperative Oncology Group Performance Status (ECOGPS):			
Usual Weight:		Current Weight:	
Nutrition:	BMI	Mini Nutritional Assessment (MNA)	
Referral:	[ ] Yes	[ ] No	
Social Worker	Geriatric Depression Scale (GDS)		Mini Mental Status Evaluation (MMSE)
Referral:	[ ] Yes	[ ] No	

_______________________________________ ___________ ___________
Physician's Signature — Time — Date

_______________________________________ ___________________
Printed Name — Pager Number

PATIENT NAME: ______________________

DATE OF BIRTH: ______________________

MR#: ______________________
